# Remanufacturing Marketing Decisions in the Presence of Retailing Platforms in the Carbon Neutrality Era

**DOI:** 10.3390/ijerph19010384

**Published:** 2021-12-30

**Authors:** Xiaojiao Qiao, Xiukun Zhao, Jinhui Zou

**Affiliations:** 1 School of Management, Tianjin University of Technology, Tianjin 300384, China; qxjiao0124@email.tjut.edu.cn; 2 School of Management Science and Engineering, Tianjin University of Finance and Economics, Tianjin 300222, China; 3 Business School, Nankai University, Tianjin 300071, China; 9820200094@nankai.edu.cn

**Keywords:** remanufacturing, marketing decision, sustainable development, supply chain management, carbon neutrality

## Abstract

Background: In response to the specific goals of carbon peaking and neutrality, remanufacture is becoming increasingly popular. With the marketplace being more and more adopted, an independent remanufacturer (IR) could sell its products via either the reselling model or the marketplace model. In order to contribute more to carbon neutrality, we investigate the optimal marketing decision for remanufacturing. We construct two models namely reselling model and the marketplace model, and further explore the effects of each marketing model on the decisions and profits of both the IR and the platform firm. Methods: We examine a platform firm that sells new products and an IR that sells remanufactured products under two marketing models based on game theory: (1) a reselling model in which the IR sells remanufactured products to the platform firm; then the platform firm resells to consumers; (2) a marketplace model in which the IR sells remanufactured products to consumers through the platform. Results: Our results show that aiming at carbon neutrality, the IR would be induced by the marketplace model to undertake remanufacturing operations and remanufacture products as many as it could still meet the market demand. Meanwhile, the marketplace model encourages the IR to rethink its work and manufacture more products under certain conditions. Furthermore, both the platform firm and the IR prefer the marketplace model to the reselling model within a Pareto zone. In addition, we find that both the platform firm and the IR could benefit from the marketplace model when they take carbon neutrality under consideration. Conclusions: This study provides managerial insight from two aspects. Remanufactures could decide their marketing model via thorough consideration of market competition, commission rate, and production cost. The government could do more to protect the marketplace environment in order to stimulate the internal vitality of the platform in the achievement march of carbon neutrality purpose. That is, this study will provide good guidance for sustainable development decision-making of remanufacturing marketing platforms, and further contributes to the achievement of the carbon neutrality goal.

## 1. Introduction

In response to global climate change, a series of specific goals and measures for carbon peaking and neutrality have been put forward. Among these, remanufacturing acting as a typical form of circular economy is getting more and more attention. During the exploration of how to contribute more for sustainable development as well as carbon neutrality, remanufacturing has realized large-scale production, which is not only an important direction of post-market transformation but also an important measure of post-marketing strategies [1]. Compared with manufacturing new products, remanufacturing significantly contributes to energy conservation and emission reduction [2]. Evidence shows that remanufacturing can save energy by 60%, materials by 70%, and costs by 50%; moreover, it hardly produces solid waste but indeed reduces air pollutant emissions by more than 80%, which is of great significance to the sustainable development of the economy and society [3]. Therefore, governments have been committed to proposing relevant policies to encourage remanufacturing activities for promoting environmental awareness or even building a resource-saving and environment-friendly society. For example, the implementation of the waste electrical and electronic equipment (WEEE) and the extended producer responsibility (EPR) policies mainly aims to achieve the goal aforementioned by requesting firms to take responsibility for their produced goods [4]. In particular, the WEEE requires all original equipment manufacturers (OEMs) to recycle and dispose of their products [5], while the EPR requires producers to bear environmental responsibility for their products throughout their life cycle [6]. Furthermore, considering the regulatory pressure of the government and the green demand of consumers [7], manufacturers enter the remanufacturing industry to expand their market scale and improve their market competitiveness [8]. The globally integrated resource recycling projects of Fuji Xerox and the international remanufacturing projects of Caterpillar are the typical projects initiated by such manufacturers, and provide useful experience for other manufacture aiming at sustainable development.

Although remanufacturing has been receiving a growing amount of attention, most related research has focused on the sustainability of remanufactured products, consumer behavior attributes, and channel competition. Very limited literature has addressed the effects of various marketing models concerning retailing platforms on the remanufacturing strategies of firms. Generally, a remanufactured product is confronted with serious problems of channel selection, platform profitability, and professional division [9]. For example, most manufacturers have advantages in market channels but perform poorly in remanufacturing technology. Conversely, some remanufacturers do not perform well in marketing channels, but they are cost-efficient [10].

In reality, the products provided by the remanufacturers often need to be sold through existing channels. For example, Caterpillar cooperates with Yuchai Company China and Land Rover UK. Caterpillar is responsible for the remanufacturing of the products sold by Yuchai Company and further, it sells remanufactured parts or whole machines through the distributors of Yuchai Company. Land Rover UK, a world-famous automobile manufacturer, does not engage in the process of remanufacturing but does sell various remanufactured products provided by Caterpillar, including spare parts to the end consumers [11]. To sum up, the firms have to decide whether to involve in remanufacturing and which marketing decision to select in their chase of sustainable development or even carbon neutrality.

Motivated by such industrial practice, we consider two types of marketing models with an independent remanufacturer (IR) selling remanufactured products and a platform firm selling new products. Furthermore, the IR has two options to sell its remanufactured products: (1) a reselling model in which the IR sells remanufactured products to the platform firm and then the platform firm resells the products to consumers via its platform (e.g., Caterpillar remanufactures the products and sells to Yuchai Company, and then Yuchai Company resells the products); (2) a marketplace model in which the IR sells remanufactured products to consumers through the platform directly (e.g., Caterpillar remanufactures the products, and directly sells them via the distributor system belongs to Yuchai Company or Land Rover UK). On the basis of the theoretical model, we seek to address the following research questions.

(1) Under the case where the IR and the platform firm compete with each other in a Nash game, what effects do the reselling and marketplace models have on the remanufacturing operations of the IR and the marketing channel preference of each partner?
(2) Under the case where the IR and the platform firm compete with each other in a Stackelberg game, how does the leader status of a platform firm affect the remanufacturing operations and marketing channel preference of each partner?

Our analyses reveal some interesting results. Firstly, in the marketplace model, the IR becomes more willing to remanufacture products when the commission rate under the marketplace model is small enough. Furthermore, the marketplace model helps to increase the number of remanufactured products under certain conditions where the commission rate is low and the production cost for the new product is moderate. That is, proper marketing decisions in remanufactures would stimulate firms to adopt a lower commission rate, and eventually contribute more to carbon neutrality. Compared with the reselling model, the commission rate has a threshold so that the platform firm and the IR can obtain higher profits under the marketplace model. In addition, under the case in which the IR and the platform firm compete with each other in a Stackelberg game, we also find that both the platform firm and the IR can benefit from the marketplace model even if the platform firm is the leader and the IR is forced to be a game follower.

This study contributes to the literature in two aspects. Firstly, we analyze an important but neglected topic in the field of remanufacturing marketing, that is, the influences of two marketing models on the remanufacturing operations of the IR and the marketing channel preference of each partner. It shows that the marketplace model may induce the IR to become more willing to undertake remanufacturing operations and consequently remanufacture more products. Meanwhile, under certain conditions, the marketplace model may benefit both the platform firm and the IR. Secondly, although some studies have investigated the platform firm as to whether being a leader or not may affect the development of the remanufacturing industry in the remanufacturing literature, production decision of remanufacturing, consumer behavior attributes, and channel competition are excluded and little is known about the influence of the platform firm with leadership on the IR in selling its remanufactured products. All the above findings would still hold regardless of whether the platform firm has leadership or not. Moreover, this work provides managerial insight from two aspects. On the one hand, remanufactures could decide their marketing model via thorough consideration of market competition, commission rate, and production cost. On the other hand, the government could do more to protect the marketplace environment in order to stimulate the internal vitality of the platform in the achievement march of carbon neutrality purpose.

The remainder of the paper is organized as follows. Section 2 reviews the related studies to position the scope of this research. Section 3 presents the theoretical models. Then, Section 4 and Section 5 derive the optimal solutions and analyses to address the research questions. Section 6 further derives the optimal solutions in a Stackelberg game where the platform firm performs as a game leader. Lastly, Section 7 provides the conclusion by discussing the main results and findings.

## 2. Literature Review

Remanufacturing has been widely explored. However, current related works have primarily focused on production decision of remanufacturing, consumer behavior attributes, and channel competition.

Study on production decision of remanufacturing mainly concerns on the balance of environmental, social, and economic factors (e.g., [12,13]), which is the sustainable and essential strategy for business growth [14]. On the one hand, some studies have attempted to determine the sustainable external influence factors to production decision of remanufacturing [15]. For example, the environmental policies of a regulator play an important role in the sustainability of remanufacturing products (e.g., [16,17,18,19]). On the other hand, related works have also explored the internal influence factors to production decision of remanufacturing, including supply chain network design for demand and return quantities (e.g., [20,21]), cooperation effectiveness of internal stakeholders [10], process innovation of remanufacturing (e.g., [22,23]) and the relationship between product quality improvement and remanufacturing [24], which has an important impact on the remanufacturing industry. Conversely, we consider remanufacturing from the perspectives of marketing channel choice in the presence of retailing platforms. Literature on marketing decisions of retail platforms [25,26] would surely make sense on the remanufacture marketing decision, however, we would not provide more since the literature body is so large and mature.

Another stream of related works is consumer behavior attributes, which is the key part of research on marketing strategies for remanufacturing [13]. Some works have explored the effect of green certification of remanufactured products on consumer perceptions [27], the trade-in marketing strategy of remanufacturing (e.g., [28,29]), and the influence factors of consumer behavior toward remanufactured products [30], such as warranty policy [31], and behavior motivation of buying remanufactured products by subsidy [32]. Other studies have investigated the impact of price and brand on consumer behavior. For instance, literature [1] showed that price discrimination is an effective marketing scheme for remanufacturing firms. Literature [33] explored the pricing and branding decisions for remanufactured fashion products. We also consider consumer behaviors, but we mainly focus on platform firms’ channel preference in the presence of reselling and marketplace models where the IR sells remanufactured products directly or indirectly.

Our work also relates to the research on channel competition under the supply chain. Some works have focused on channel competition but still face challenges in the new digital operation era. On the one hand, the channel competition between remanufacturing and new products is always the focal point of studies. For example, some research (e.g., [9,34,35,36,37]) explored the impact of remanufactured products on new products and the two-sided competition in different channel structures in the supply chain. On the other hand, firms have increasingly been interested in secondary markets and platform operations. Literature [8] illustrated the competitive advantage of firms by remanufacturing and selling remanufactured products in a secondary market. Literature [2] showed the effects of secondary markets on the pricing, trade-in remanufacturing, and entry decisions of OEMs using a two-period model. Literature [38] investigated the contrast and assimilation effects of competitive remanufacturing and pricing strategy on OEMs and third-party platforms. Literature [39] explored the impacts of green innovation and channel service in a dual-channel value chain. In contrast to the above work, we consider the channel competition between the reselling model and marketplace model. Furthermore, these two channels are used to sell remanufactured products and the results discover that the marketplace model may dominate the reselling model under certain conditions, regarding inducing the IR to undertake remanufacturing operations, remanufacturing more products, and improving both firms’ profits.

## 3. Model

**Figure 1:**
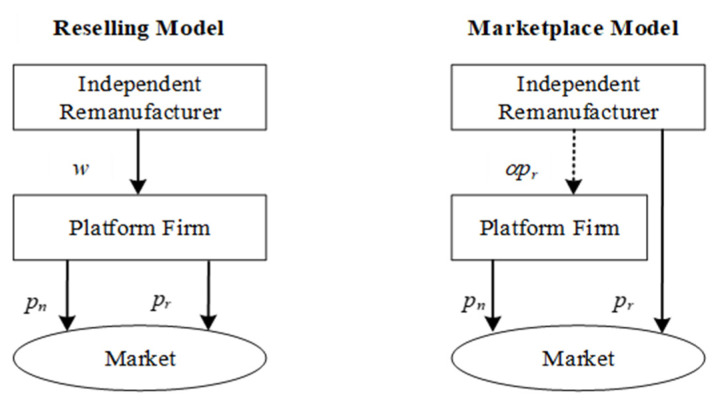
Structure of two marketing models.

In this study, we consider a platform firm that operates a retailing platform and sells new products to consumers via the platform. Then, we examine an IR that undertakes remanufacturing and sells remanufactured products to consumers via the retailing platform. The two types of marketing models are provided by the platform firm. Under reselling model with which the IR sells remanufactured products to the platform firm at a wholesale price *w*; then, the platform firm resells these products at $p_{r}$ (if remanufactured) or $p_{n}$ (if new) to consumers via its platform. While under marketplace model the IR can sell remanufactured products at $p_{r}\;$ to consumers through the platform directly. However, a commission fee needs to be paid by the IR. The commission rate is α, α ranges from 0 to 1, and the corresponding commission fee is α$p_{r}$. The structures of these two marketing models are presented in Figure 1. We use $c_{n}$ and $c_{r}$ to denote the production costs for each new product and each remanufactured product, respectively. In addition, $c_{n}$ and $c_{r}$ satisfy $c_{n}\;$>$\;c_{r}\;$≥ 0. To focus on the insightful cases in which the IR has incentives to undertake remanufacturing operations and the sale of remanufactured products is positive, we normalize $c_{r}$ to 0. The sequence of events is as follows. Firstly, the platform firm decides which marketing model should be applied to the IR. Under reselling model, the IR determines whether to accept the model and once accept decide the wholesale price further, and finally the platform firm decides the quantities of new and remanufactured products. Otherwise, that is, under the marketplace model, the platform firm decides the quantity of the new product, and the IR simultaneously determines the quantity of the remanufactured product. Here, we consider a Nash game between the platform firm and the IR under the marketplace model. We will further explore a Stackelberg game where the platform firm acts as leader followed by the IR when the IR adopts the marketplace model.

Regarding the demand for new and remanufactured products in the market, we assume that consumers are heterogeneous in their willingness to pay for the new product. Following the previous research in operations management on remanufacturing, we assume that the willingness of consumers to pay for a unit of the new product is *v*, which is uniformly distributed in (0,1). Without loss of generality, we normalize the potential market size to 1. The willingness of consumers to pay for a unit of the remanufactured product is a fraction of that of the new product, which is denoted by *θ*. 0 ≤ $c_{r}$ < *θ* ≤ 1, thus ensuring that the firm could benefit from remanufacture. Therefore, the surplus a consumer obtains from consuming a unit of the new product is $v-p_{n}$, and the respective surplus that a consumer acquires from consuming a unit of the remanufactured product is $\theta v-p_{r}$. Consumers make their purchasing decisions by maximizing the surplus they gain from consuming the new and remanufactured products. By comparing $v-p_{n}$ and $\theta v-p_{r}$, we can obtain the demand for the new and remanufactured products, which are expressed by $q_{n}=\int_{\frac{p_{n}-p_{r}}{1-\theta }}^{1}1dv$ and $q_{r}=\int_{\frac{p_{r}}{\theta }}^{\frac{p_{n}-p_{r}}{1-\theta }}1dv$.

Furthermore, we can derive the inverse demand functions of the new and remanufactured products, which are expressed by Equations (1) and (2) below.

(1) $$p_{n}=1-q_{n}-\theta q_{r}$$

(2) $$p_{r}=\theta \left(1-q_{n}-q_{r}\right)$$

## 4. Equilibrium Analysis

In this section, we first derive the optimal decisions of the platform firm and the IR under the reselling model. Then, we obtain the result under the marketplace model. Finally, we provide some comparative analysis in terms of the optimal quantities of the new and remanufactured products as well as the optimal profits.

### 4.1. Reselling Model

Under the reselling model (denoted by R), the IR first sells the remanufactured product to the platform firm at w. Then, the platform firm sells new and remanufactured products via its platform to consumers. In this case, the IR first determines the wholesale price of the remanufactured product, then the platform firm decides the quantities of the new and remanufactured products. On the basis of Equations (1) and (2) in Section 3, we can obtain the profit functions of the IR and the platform firm, which are respectively given below.

(3) $$\pi_{IR}^{R}\left(w\right)=\left(w-c_{r}\right)q_{r},$$

(4) $$\pi_{F}^{R}\left(q_{n},q_{r}\right)=(p_{n}-c_{n})q_{n}+\left(p_{r}-w\right)q_{r}$$

**Proposition 1.**  *Under the reselling model, the optimal wholesale price and the quantities of the new and remanufactured products are:*

*(1)*  *If* $c_{n}<\frac{c_{r}}{\theta }$*,then* $w^{R*}=\frac{c_{r}}{\theta }$*,*$q_{n}^{R*}=\frac{1-c_{n}}{2}$ *and* $q_{r}^{R*}=0$*;*
*(2)*  *If* ${\hat{c}}_{n}\geq c_{n}\geq \frac{c_{r}}{\theta }$*,then* $w^{R*}=\frac{\theta c_{n}+c_{r}}{2}$*,*$q_{n}^{R*}=\frac{2-2\theta -\left(2-\theta \right)c_{n}+c_{r}}{4\left(1-\theta \right)}$ *and* $q_{r}^{R*}=\frac{\theta c_{n}-c_{r}}{4\theta \left(1-\theta \right)}$*;*
*(3)*  *If* $c_{n}>{\hat{c}}_{n}$*,then* $w^{R*}=\frac{1+\theta -c_{n}+c_{r}}{2}$*and* $q_{n}^{R*}=q_{r}^{R*}=\frac{1+\theta -c_{n}-c_{r}}{4\left(1+3\theta \right)}$*,where* ${\hat{c}}_{n}=\frac{\sqrt{\left(1-\theta \right)\theta }\left(1+\theta -c_{r}\right)+\sqrt{1+3\theta }c_{r}}{\theta \sqrt{1+3\theta }+\sqrt{\left(1-\theta \right)\theta }}$.

*Proof is in Appendix A.*

Proposition 1 shows that three cases in which the procurement of remanufactured products will be 0, less than, or equal to that of the new products, respectively. This result mainly relies on the production cost of the new product. In particular, when the production cost of the new product is small, the platform firm will not procure any remanufactured products from the IR. The cost advantage of the remanufactured product is high enough to ensure that reselling remanufactured products could bring a positive marginal profit to the platform firm.

An interesting result is that the high cost of the new product does not certainly increase the wholesale price of the remanufactured product. Intuitively, the higher the production cost of the new product, the stronger the cost advantage of the remanufactured product. As a result, the platform firm prefers to procure more remanufactured products from the IR. Therefore, the IR should increase the wholesale price of the remanufactured product. However, we can observe that the wholesale price decreases with the cost of the new product when the production cost of the new product is high. The increase in the production cost leads to a significantly decreased supply of the new product when the production cost of the new product is high. To coordinate the competition between the new and the remanufactured products and obtain a high margin profit, the platform firm tends to reduce the procurement of the remanufactured product. However, from the perspective of the IR, the optimal option is to reduce the wholesale price to encourage the platform firm to procure more the remanufactured product. Therefore, under the reselling model, from the perspective of sustainable development, the core issue of renewable energy promotion focusing on remanufacturing lies in production cost and transaction cost in the achieving of carbon neutrality goal.

### 4.2. Marketplace Model

Under the marketplace model (denoted by M), the IR sells remanufactured products via the platform providing by the platform firm and pays a commission fee for each unit remanufactured correspondingly. At the same time, the platform firm sells new products to consumers as well. In this case, the platform firm and the IR simultaneously decide the quantity of both the new product and the remanufactured product. Similarly, we can obtain the profit functions of the platform firm and the IR, which are respectively expressed by

(5) $$\pi_{F}^{M}\left(q_{n}\right)=(p_{n}-c_{n})q_{n}+\alpha p_{r}q_{r}$$

(6) $$\pi_{IR}^{M}\left(q_{r}\right)=\left(\left(1-\alpha \right)p_{r}-c_{r}\right)q_{r}$$

$$0\leq q_{r}\leq q_{n}.$$

**Proposition 2.**  *Under the marketplace model, the optimal quantities of the new and remanufactured products are:*

*(1)*  *If* $c_{n}<{\bar{c}}_{n}\left(\alpha \right)$*,then* $q_{r}^{M*}=0$ *and* $q_{n}^{M*}=\frac{1-c_{n}}{2}$*;*
*(2)*  *If* ${\bar{c}}_{n}\left(\alpha \right)\leq c_{n}\leq {\bar{c}}_{n}\left(\alpha \right)$*,then* $q_{r}^{M*}=\frac{\left(1-\alpha \right)\theta \left(1+c_{n}\right)-2c_{r}}{\left(1-\alpha \right)\theta \left(4-\theta -\alpha \theta \right)}$ *and* $q_{n}^{M*}=\frac{\left(1-\alpha \right)\left(2-\theta -\alpha \theta -2c_{n}\right)-\left(1+\alpha \right)c_{r}}{\left(1-\alpha \right)\theta \left(4-\theta -\alpha \theta \right)}$*;*
*(3)*  *If* $c_{n}>{\tilde{c}}_{n}\left(\alpha \right)$*,then* $q_{r}^{M*}=q_{n}^{M*}=\frac{1-c_{n}}{2+\theta +\alpha \theta }$*,where* ${\tilde{c}}_{n}\left(\alpha \right)=\frac{\left(1-\alpha \right)\theta -\left(1-\alpha^{2}\right)\theta^{2}+\left(2+\theta +\alpha \theta \right)c_{r}}{3\left(1-\alpha \right)\theta }$ *and* ${\bar{c}}_{n}\left(\alpha \right)=\frac{2c_{r}}{\left(1-\alpha \right)\theta }-1$.
  *Furthermore,*${\bar{c}}_{n}\left(\alpha \right)$ *is increasing in* α*,while* ${\tilde{c}}_{n}\left(\alpha \right)$ *is decreasing in*α *when* $\alpha <1-\frac{\sqrt{2\left(1+\theta \right)c_{r}}}{\theta }$.

*Proof is in Appendix A.*

Proposition 2 shows that all scenarios under reselling model also exist under the case where the platform firm offers a marketplace model. It would be easily found that the commission rate plays an important role in remanufacturing operations of the IR. In particular, with the increase of the commission fee charged by the platform firm, the cost of remanufacturing also increases, and the IR becomes less willing to engage in remanufacturing. Nevertheless, with the rise of the production cost of the new product, the IR gains a higher cost advantage in remanufacturing. In this case, the increase of the commission rate induces the IR to engage in remanufacturing and increase the quantity of the remanufactured product as illustrated by Proposition 3. When the cost of the new product goes higher, the quantity of the new product would decrease tremendously. Further, a higher commission rate increases the likelihood that the quantity of the remanufactured product is equal to that of the new product. Therefore, under the marketplace model, IR should focus on the cost advantage of remanufactured products by improving the efficiency of the manufacturing process, accelerating the automation and intelligent transformation of the production line, and ensuring the use of clean energy.

**Proposition 3.**  *Under the marketplace model, the effect of the commission rate on the optimal quantities of the new and remanufactured products are as follows:*

*(1)*  *If* $c_{n}<{\bar{c}}_{n}\left(\alpha \right)$*,then* $\frac{dq_{r}^{M*}}{d\alpha }=\frac{dq_{n}^{M*}}{d\alpha }=0$*;*
*(2)*  *If* ${\bar{c}}_{n}\left(\alpha \right)\leq c_{n}\leq {\tilde{c}}_{n}\left(\alpha \right)$*,then* $\frac{dq_{r}^{M*}}{d\alpha }>0$*if*$1-\frac{2c_{r}+2\sqrt{c_{r}\left(\left(2-\theta \right)\left(1+c_{n}\right)+c_{r}\right)}}{\theta \left(1+c_{n}\right)}>\alpha$ *and* $\frac{dq_{n}^{M*}}{d\alpha }<0$*;*
*(3)*  *If*$c_{n}>{\tilde{c}}_{n}\left(\alpha \right)$*,then* $\frac{dq_{r}^{M*}}{d\alpha }=\frac{dq_{n}^{M*}}{d\alpha }<0$.

*Proof is in Appendix A.*

Proposition 3 demonstrates how the commission rate affects the optimal decisions of the platform firm and the IR. In particular, when the production cost is low, the IR does not remanufacture any products. As a result, the quantities of the new and remanufactured products are independent of the commission rate. However, when the production cost increases, the IR remanufactures some used products. In this case, the cost advantage of remanufacturing becomes more significant since the production cost increases. Therefore, the IR prefers to increase the number of remanufactured products with the increase of the commission rate. However, when the production cost exceeds the threshold value, i.e., ${\tilde{c}}_{n}\left(\alpha \right)$, all new products sold by the platform firm in the market will be remanufactured by the IR. Therefore, the quantity of the remanufactured products has the same monotonicity as that of the new product. With the increase of the commission rate, the IR increases the price of the remanufactured product. Consequently, the platform firm raises the price of the new product and decreases the demand for the new product. Thus, under the marketplace model, the platform plays a very important role in remanufacturing the market. Moreover, proper platform activity also would benefit the sustainable development of low-carbon, energy-saving, and environmental protection industries.

## 5. Comparative Analysis

This section may be divided into subheadings. It should provide a concise and precise description of the experimental results, their interpretation, as well as the experimental conclusions that can be drawn. In this section, we provide some comparisons regarding the results under these two models. Firstly, we can obtain Proposition 4 in terms of the likelihood of the IR engaging in remanufacturing.

**Proposition 4.**  *Compared with the reselling model, the marketplace model is more likely to induce the IR to undertake remanufacturing operations and remanufacture all products when the commission rate is small enough. Mathematically,* ${\bar{c}}_{n}\left(\alpha \right)<\frac{c_{r}}{\theta }$*and*${\tilde{c}}_{n}\left(\alpha \right)<{\hat{c}}_{n}$*when*$\alpha <\min\left\{\frac{\theta -c_{r}}{\theta +c_{r}},\tilde{\alpha }\right\}$*, where*$\tilde{\alpha }$*satisfies*${\tilde{c}}_{n}\left(\alpha \right)={\hat{c}}_{n}$.

*Proof is in Appendix A.*

Proposition 4 illustrates that the IR is more willing to adopt remanufacture strategy as long as the commission rate under the marketplace model is small enough. If the commission rate is low, then the respective cost of selling the remanufactured product is also low. Therefore, the IR has strong motivation to undertake to remanufacture. However, the double marginalization effect leads to a higher reselling costs of under the reselling model. When the production cost of the new product is low, the platform firm is not willing to sell the remanufactured product even though the remanufacturing is profitable to the IR. Furthermore, the result reveals that merely providing remanufactured products under the marketplace model will more likely happen when the commission rate is low enough. The cost of selling remanufactured products is inferred to be low when the commission rate is low enough. In this case, the IR is more likely to engage in remanufacturing and remanufacturing a high quantity of the remanufactured product, especially when the production cost of the new product is high. Meanwhile, from the perspective of sustainable development, the government should standardize the marketplace model in the presence of retailing platforms in order to achieve the goal of carbon neutrality based on digital economy and platform economy.

**Proposition 5.**  *Compared with the reselling model, the marketplace model induces the IR to remanufacture more products (i.e.,* $q_{r}^{R*}<q_{r}^{M*}$ *) under the following cases:*

*(1)*  *when* ${\bar{c}}_{n}\left(\alpha \right)<c_{n}<\frac{c_{r}}{\theta }$ *and* $\alpha <\frac{\theta -c_{r}}{\theta +c_{r}}$;
*(2)*  *when* $max\left\{{\bar{c}}_{n}\left(\alpha \right),c_{r}/\theta \right\}<c_{n}<min\left\{\frac{\left(1-\alpha \right)\theta \left(4-4\theta \right)-\left(4\left(1-\alpha \right)-\left(7+\alpha^{2}\right)\theta \right)c_{r}}{\left(3-\alpha \right)\left(1-\alpha \right)\theta^{2}},\;{\tilde{c}}_{n}\left(\alpha \right),{\hat{c}}_{n}\right\}$ *;*
*(3)*  *when*${\tilde{c}}_{n}\left(\alpha \right)<c_{n}<min\left\{{\hat{c}}_{n},\frac{4\left(1-\theta \right)\theta +\left(2+\theta +\alpha \theta \right)c_{r}}{\theta \left(6-\left(3-\alpha \right)\theta \right)c_{n}}\right\}$ and $\alpha <\tilde{\alpha }$;
*(4)*  *when*$max\left\{{\tilde{c}}_{n}\left(\alpha \right),{\hat{c}}_{n}\right\}<c_{n}<\frac{2+\theta \left(9-\alpha -\theta -\alpha \theta \right)+\left(2+\theta +\alpha \theta \right)c_{r}}{2+\left(11-\alpha \right)\theta }$.

*Proof is in Appendix A.*

Proposition 5 shows that the marketplace model works in increasing the quantity of the remanufactured product under some conditions. Such status appears when the commission rate is low, and the production cost of the new product is moderate. This result is intuitive and can be easily understood. Low commission rate leads to the low cost of selling remanufactured products. Therefore, the IR is more willing to remanufacture more products. Considering that the supply of remanufactured products is restricted by the sale of the new products, the platform firm prefers to sell more new products when its production cost is moderate. Thus, the IR can remanufacture more products than that reselling models. This result also shows that firms must acquire the right to decide the sale quantities. Under the marketplace model, the platform firm and the IR determine the quantities of the new and remanufactured products, respectively. Therefore, the IR has more incentive to remanufacture more products. However, under the reselling model, the platform firm can simultaneously decide the quantities of the new and remanufactured products. To alleviate the cannibalization effect, the quantities of the new and the remanufactured are limited, especially when the production cost of the new product is relatively low at the respective range.

**Proposition 6.**  *Compared with the reselling model, a threshold of commission rate*$\hat{\alpha }$*and a cost threshold*${\tilde{c}}_{n}\left(\hat{\alpha }\right)$*exists so that the platform firm and the IR can obtain higher profit under the marketplace model when*${\tilde{c}}_{n}\left(\hat{\alpha }\right)>c_{n}>max\left\{{\tilde{c}}_{n}\left(\alpha \right),{\hat{c}}_{n}\right\}$*and*$\alpha <\hat{\alpha }$.

*Proof is in Appendix A.*

**Figure 2:**
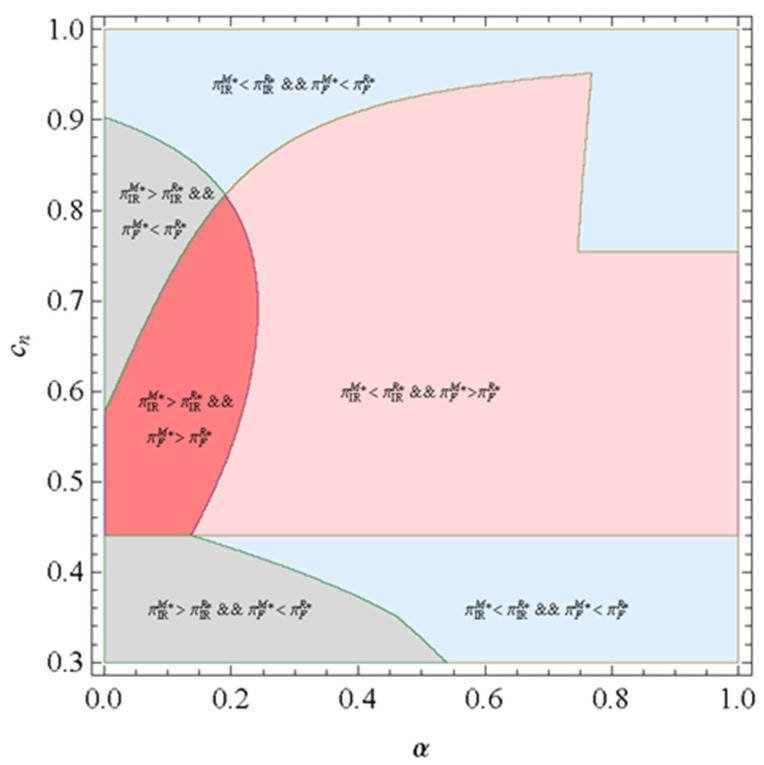
Profits of firm and IR.

Proposition 6 implies that a Pareto zone exists where the platform firm and the IR prefer the marketplace model to the reselling model. The selling of remanufactured products cannibalizes the market of the new product; however, the platform firm would get some compensation when the IR sells more remanufactured products driven by a lower commission rate. In particular, under the case where all the products sold by the platform firm are remanufactured by the IR, the cost disadvantage of the new product is relatively low if the production cost of the new product is also low. As a result, the sale of the new product is large, and all products are remanufactured and resold by the IR via the platform. Therefore, the loss caused by the cannibalization effect is smaller than the corresponding gain. In this case, both the platform firm and the IR benefit more from the marketplace model than that of the reselling model. To understand the result presented in Proposition 6 better, we further provide Figure 2, where $c_{r}$ = 0.2 and θ = 0.9. α ranges from 0 to 1, while $c_{n}$ ranges from 0.3 to 1, as it should be greater than $c_{r}$. As shown in Figure 2, firstly, the platform firm can always obtain higher profit under the reselling model with lower production costs for new products. The platform firm and the IR can always obtain higher profit under reselling model with higher production costs for new products. Secondly, the platform firm can always obtain higher profit under the marketplace model with moderate production costs for new products. Thirdly, we eventually find a region in which the commission rate is low, and the production cost of the new product is moderate. Thus, the platform firm and the IR can obtain a higher profit under the marketplace model in which the platform firm is the leader that is in line with Proposition 6.

Finally, Proposition 5 and Proposition 6 also show that, compared with the reselling model, the marketplace model in the presence of a Stackelberg game provides firms with a more suitable opportunity for their marketing decision and remanufactured products promotion. The remanufacturing industry together with the platform economy will build a foundation for energy conservation and sustainable industrial development considering the leader status of a platform firm and the goal of carbon neutrality.

## 6. Model Extensions

In Section 4, we investigate the strategy of the platform firm under the case where a Nash game occurs between the platform firm and the IR when the IR adopts the marketplace model. However, some firms (either manufacturer or retailer) that operate platforms become increasingly powerful and act as game leader when competing against the IR. Here, we investigate whether the leadership changes the results derived above. Considering reselling model, the platform firm first decides the procurement of remanufactured products and the sale of new products. Then, the IR determines the wholesale price of the remanufactured product. The profit functions of the platform firm and the IR are identical to those in the basic model. That is,

(7) $$\begin{array}{c} \pi_{F}^{RS}\left(q_{n},q_{r}\right)=\left(p_{n}-c_{n}\right)q_{n}+\left(p_{r}-w\right)q_{r}\\ 0\leq q_{r}\leq q_{n}, \end{array}$$

(8) $$\pi_{IR}^{RS}\left(w\right)=\left(w-c_{r}\right)q_{r}.\;$$

Here, we use RS to denote the reselling model with a Stackelberg game. By employing the backward induction, we can obtain the optimal decisions, which are expressed by Proposition 7 below.

**Proposition 7.**  *When a Stackelberg game occurs under the reselling model, the optimal decisions of the platform firm and the IR are*

*(i)*  *If* $c_{r}/\theta >c_{n}$*,* *then* $q_{n}^{RS*}=\frac{1-c_{n}}{2}$*,*$q_{r}^{RS*}=0$*,* *and* $w^{RS*}=\frac{\phi c_{n}+3c_{r}}{4}$*;*
*(ii)*  *If*$\frac{\left(1+\theta \right)c_{r}+\left(2-\theta \right)\theta }{3\theta }>c_{n}>c_{r}/\theta$*,* *then* $q_{n}^{RS*}=\frac{2-\theta -2c_{n}+c_{r}}{4-2\theta }$*,* $q_{r}^{RS*}=\frac{\theta c_{n}-c_{r}}{4\theta -2\theta^{2}}$*,*  *and* $w^{RS*}=\frac{\theta c_{n}+\left(3-2\theta \right)c_{r}}{4-2\theta }$ *;*
*(iii)*  *If*$c_{n}>\frac{\left(1+\theta \right)c_{r}+\left(2-\theta \right)\theta }{3\theta }$*,then*$q_{n}^{RS*}=\frac{1+\theta -c_{n}-c_{r}}{2+8\theta }$*,*$q_{r}^{RS*}=\frac{1+\theta -c_{n}-c_{r}}{2+8\theta }$*,and*$w^{RS*}=\frac{\phi \left(3\theta +c_{n}\right)+\left(3+13\theta \right)c_{r}}{4+16\theta }$.

*Proof is in Appendix A.*

Similarly, depending on the value range of $c_{n}$, three cases appear. Cases are no remanufacturing, partial remanufacturing and full remanufacturing.

Considering the marketplace model, we can obtain the profit functions of the platform firm and the IR below.

(9) $$\pi_{F}^{MS}\left(q_{n}\right)=(p_{n}-c_{n})q_{n}+\alpha p_{r}q_{r}$$

(10) $$\pi_{IR}^{MS}\left(q_{r}\right)=\left(\left(1-\alpha \right)p_{r}-c_{r}\right)q_{r}$$

$$0\leq q_{r}\leq q_{n}.$$

Here, we use MS to denote the marketplace model with a Stackelberg game.

By using the backward induction approach, we can derive the optimal decisions of the platform firm and the IR, which are summarized by the proposition below.

**Proposition 8.**  *When a Stackelberg game occurs under the marketplace model, the optimal decisions of the platform firm and the IR are presented by*Table 1.

*Proof is in Appendix A.*

Proposition 8 demonstrates that the platform firm has more choices than the IR to set its optimal decisions of the quantity of the new product. However, the IR still implements three types of remanufacturing strategies. In this case, the leadership enables the platform firm to perform make more flexible than that under the Nash game case. In addition, depending on the value of the commission rate, either no remanufacturing or full remanufacturing at the boundary point will be the optimal response of the IR.

Then we explore whether the marketplace model benefits the platform firm and the IR even though it forces the IR to be a game follower, and we get Proposition 9.

**Proposition 9.**  *Compared with the reselling model, a threshold of the commission rate $\hat{\bar{\alpha }}$ and a cost threshold*${\tilde{\bar{c}}}_{n}\left(\hat{\bar{\alpha }}\right)$*exists so that the platform firm and the IR can obtain higher profit under the marketplace model when* $\;max\left\{{\tilde{\bar{c}}}_{n}\left(\alpha \right),\frac{\left(1-\alpha \right)\theta \left(1-\left(2+\alpha \right)\theta \right)+2\left(1+\theta +2\alpha \theta \right)c_{r}}{3\left(1-\alpha \right)\theta },\frac{\left(1+\theta \right)c_{r}+\left(2-\theta \right)\theta }{3\theta }\right\}<c_{n}<{\tilde{\bar{c}}}_{n}\left(\hat{\bar{\alpha }}\right)$*and* $\alpha <\hat{\bar{\alpha }}$.

*Proof is in Appendix A.*

Similar to the result under the Nash game, Proposition 9 illustrates that both the platform firm and the IR could benefit from the marketplace model even if the platform firm is the leader and the IR acts as follower. As long as the commission rate is low enough, the marketplace model, which allows the IR to decide the quantity of the remanufactured product, can help the IR obtain a higher profit than that under the reselling model. Therefore, a Pareto zone exists in which the platform firm and the IR prefer the marketplace model to the reselling model.

To sum up, remanufacturing marketing achieves more market demand through the marketplace model in the presence of retailing platforms compared with the reselling model. There are more ways to achieve carbon neutrality for the energy conservation and emission reduction industry similar to remanufacturing under marketplace model.

## 7. Conclusions

Remanufacturing increasingly has concerns as a significant measure for carbon peaking and neutrality, and has gained lots of progress. In practice, many firms and IRs, such as Yuchai Company China, Land Rover UK and Caterpillar, have adopted different channels to sell remanufactured products, however, in theory, very little research has discussed the integration influence of the channel choice and the leadership on the marketing channel preference of each partner. In order to make appreciate use of remanufacturing in the achievement of carbon neutrality, we investigate the remanufacturing marketing decision in the presence of retailing platform. On the one hand, we study the impact of the reselling model and marketplace model on the remanufacturing operations of the IR and the marketing channel preference of each partner. On the other hand, we explore the effects of the leadership of the platform firm in the marketplace model on the remanufacturing operations and marketing channel preference of each partner.

To obtain management insights concerning marketing models for retailing platforms with remanufacturing, we discuss the optimal strategies of both the platform firm and the IR and obtain some results. Firstly, the marketplace model is more likely induce the IR to undertake remanufacturing. As remanufacturing is a typical way to save energy and is environment friendly, it would motivate the government to propose proper policy making marketplace model that is easier to adopt. In the past decades, China kept building a regulatory system conducive to the orderly growth of the platform economy. Secondly, the marketplace model induces the IR to remanufacture more products. This is great progress after the IR adopted the marketplace model—they remanufacture products and remanufacture more. When IR realizes large-scale remanufacture production it would certainly lead to more contribution to the achievement of carbon neutrality. Thirdly, a Pareto zone exists where the platform firm and the IR prefer the marketplace model to the reselling model. This means a lot to the resource allocation of a certain firm. That is, within the Pareto zone, the IR focus on the process of remanufacturing while the platform concentrates on its development of distribution system. Additionally, the leadership enables the platform firm to make more flexible choices. Both the platform firm and the IR could benefit from the marketplace model. Therefore, the manufactures could rethink their remanufacture marketing decision via a certain aspect they do care about, and the government would concentrate on how to manage the marketplace environment to promote the contribution made by the platform in the process of carbon neutrality achievement.

Our discussion is subject to three limitations. Firstly, we assume a platform firm and an IR with complete information, both of which can be relaxed in future research. Secondly, we assume that the consumers are homogeneous under the two marketing models. In fact, the attributes of consumers include two dimensions: strategic consumer and myopic customers. Thirdly, we do not discuss the competition between the supply chains under the two marketing models in this study. However, such discussion may be an important direction for future research.

In summary, we expand the research on remanufacturing focusing on consumer behavior attributes, and channel competition. Indeed, we provide two marketing models to consider how different marketing channels affect the profitability of the platform firm and the IR. We also put forward valuable insights into the importance of channel preference and leadership on the profitability of the platform firm and the IR, which is not only beneficial to the platform firm and the IR but also beneficial to sustainable collaborative governance of the supply chain and carbon neutrality achievement of countries in the remanufacturing field.

## Figures and Tables

**Table 1:** Optimal decisions of platform firm and IR under marketplace model with Stackelberg game.

Conditions		$q_{n}^{MS*}$	$q_{r}^{MS*}$
*Case 1:* $\;\alpha >\frac{\theta -c_{r}}{\theta +c_{r}}$	$c_{n}\leq {\overset{=}{c}}_{n}\left(\alpha \right)$	$\frac{1-c_{n}}{2}$	0
	${\overset{=}{c}}_{n}\left(\alpha \right)<c_{n}<\frac{\left(1-\alpha \right)\theta \left(2-\theta -2\alpha \theta \right)+\left(4+\left(1-\alpha \right)\theta \right)c_{r}}{6\left(1-\alpha \right)\theta }$	$\frac{\left(1-\alpha \right)\left(2-\theta -\alpha \theta -2c_{n}\right)+c_{r}}{\left(1-\alpha \right)\left(4-\left(2+\alpha \right)\theta \right)}$	$\frac{\left(1-\alpha \right)\theta \left(2-\theta +2c_{n}\right)-\left(4-\theta -\alpha \theta \right)c_{r}}{2\left(1-\alpha \right)\theta \left(4-\left(2+\alpha \right)\theta \right)}$
	$\frac{\left(1-\alpha \right)\theta \left(2-\theta -2\alpha \theta \right)+\left(4+\left(1-\alpha \right)\theta \right)c_{r}}{6\left(1-\alpha \right)\theta }\leq c_{n}\leq \;\frac{\left(1-\alpha \right)\theta \left(1-\left(2+\alpha \right)\theta \right)+2\left(1+\theta +2\alpha \theta \right)c_{r}}{3\left(1-\alpha \right)\theta }$	$\frac{1}{3}-\frac{c_{r}}{3\left(1-\alpha \right)\theta }$	$\frac{1}{3}-\frac{c_{r}}{3\left(1-\alpha \right)\theta }$
	$c_{n}>\frac{\left(1-\alpha \right)\theta \left(1-\left(2+\alpha \right)\theta \right)+2\left(1+\theta +2\alpha \theta \right)c_{r}}{3\left(1-\alpha \right)\theta }$	$\frac{1+\alpha \theta -c_{n}}{2+2\theta +4\alpha \theta }$	$\frac{1+\alpha \theta -c_{n}}{2+2\theta +4\alpha \theta }$
*Case 2*: $\frac{\theta -c_{r}}{\theta +c_{r}}\geq \alpha \;\geq \frac{\theta -c_{r}}{\theta +3c_{r}}$	$c_{n}\leq \frac{2c_{r}}{\left(1-\alpha \right)\theta }-1$	$\frac{1-c_{n}}{2}$	0
	$\frac{2c_{r}}{\left(1-\alpha \right)\theta }-1<c_{n}<\frac{\left(4-\theta -\alpha \theta \right)c_{r}-\left(1-\alpha \right)\left(2-\theta \right)\theta }{2\left(1-\alpha \right)\theta }$	$1-\frac{c_{r}}{\left(1-\alpha \right)\theta }$	0
	$\frac{\left(4-\theta -\alpha \theta \right)c_{r}-\left(1-\alpha \right)\left(2-\theta \right)\theta }{2\left(1-\alpha \right)\theta }\leq c_{n}\leq \frac{\left(1-\alpha \right)\theta \left(2-\theta -2\alpha \theta \right)+\left(4+\left(1-\alpha \right)\theta \right)c_{r}}{6\left(1-\alpha \right)\theta }$	$\frac{\left(1-\alpha \right)\left(2-\theta -\alpha \theta -2c_{n}\right)+c_{r}}{\left(1-\alpha \right)\left(4-\left(2+\alpha \right)\theta \right)}$	$\frac{\left(1-\alpha \right)\theta \left(2-\theta +2c_{n}\right)-\left(4-\theta -\alpha \theta \right)c_{r}}{2\left(1-\alpha \right)\theta \left(4-\left(2+\alpha \right)\theta \right)}$
	$\frac{\left(1-\alpha \right)\theta \left(2-\theta -2\alpha \theta \right)+\left(4+\left(1-\alpha \right)\theta \right)c_{r}}{6\left(1-\alpha \right)\theta }<c_{n}<\;\frac{\left(1-\alpha \right)\theta \left(1-\left(2+\alpha \right)\theta \right)+2\left(1+\theta +2\alpha \theta \right)c_{r}}{3\left(1-\alpha \right)\theta }$	$\frac{1}{3}-\frac{c_{r}}{3\left(1-\alpha \right)\theta }$	$\frac{1}{3}-\frac{c_{r}}{3\left(1-\alpha \right)\theta }$
	$c_{n}\geq \frac{\left(1-\alpha \right)\theta \left(1-\left(2+\alpha \right)\theta \right)+2\left(1+\theta +2\alpha \theta \right)c_{r}}{3\left(1-\alpha \right)\theta }$	$\frac{1+\alpha \theta -c_{n}}{2+2\theta +4\alpha \theta }$	$\frac{1+\alpha \theta -c_{n}}{2+2\theta +4\alpha \theta }$
*Case 3*: $\;\alpha <\frac{\theta -c_{r}}{\theta +3c_{r}}$	$\frac{2c_{r}}{\left(1-\alpha \right)\theta }-1$	$\frac{1-c_{n}}{2}$	0
	$\frac{2c_{r}}{\left(1-\alpha \right)\theta }-1<c_{n}<\frac{\left(4-\theta -\alpha \theta \right)c_{r}-\left(1-\alpha \right)\left(2-\theta \right)\theta }{2\left(1-\alpha \right)\theta }$	$1-\frac{c_{r}}{\left(1-\alpha \right)\theta }$	0
	$\frac{\left(4-\theta -\alpha \theta \right)c_{r}-\left(1-\alpha \right)\left(2-\theta \right)\theta }{2\left(1-\alpha \right)\theta }\leq c_{n}\leq {\tilde{\bar{c}}}_{n}\left(\alpha \right)$	$\frac{\left(1-\alpha \right)\left(2-\theta -\alpha \theta -2c_{n}\right)+c_{r}}{\left(1-\alpha \right)\left(4-\left(2+\alpha \right)\theta \right)}$	$\frac{\left(1-\alpha \right)\theta \left(2-\theta +2c_{n}\right)-\left(4-\theta -\alpha \theta \right)c_{r}}{2\left(1-\alpha \right)\theta \left(4-\left(2+\alpha \right)\theta \right)}$
	${\tilde{\bar{c}}}_{n}\left(\alpha \right)<c_{n}$	$\frac{1+\alpha \theta -c_{n}}{2+2\theta +4\alpha \theta }$	$\frac{1+\alpha \theta -c_{n}}{2+2\theta +4\alpha \theta }$

${\overset{=}{c}}_{n}\left(\alpha \right)$ satisfies; $\frac{{\left(1-c_{n}\right)}^{2}}{4}=\frac{\left({\left(1-\alpha \right)}^{2}\theta \left({\left(2-\theta \right)}^{2}-4c_{n}\left(2-\theta -\alpha \theta -c_{n}\right)\right)+2\left(1-\alpha \right)\theta \left(2-\theta -\alpha \theta -2c_{n}\right)c_{r}-\left(4\alpha -{\left(1+\alpha \right)}^{2}\theta \right)c_{r}^{2}\right)}{4{\left(1-\alpha \right)}^{2}\theta \left(4-\left(2+\alpha \right)\theta \right)}$, and ${\tilde{\bar{c}}}_{n}\left(\alpha \right)$ satisfies; $\frac{{\left(1+\alpha \theta -c_{n}\right)}^{2}}{4\left(1+\theta +2\alpha \theta \right)}=\frac{\;\left({\left(1-\alpha \right)}^{2}\theta \left({\left(2-\theta \right)}^{2}-4c_{n}\left(2-\theta -\alpha \theta -c_{n}\right)\right)+2\left(1-\alpha \right)\theta \left(2-\theta -\alpha \theta -2c_{n}\right)c_{r}-\left(4\alpha -{\left(1+\alpha \right)}^{2}\theta \right)c_{r}^{2}\right)}{4{\left(1-\alpha \right)}^{2}\theta \left(4-\left(2+\alpha \right)\theta \right)}$.

## Appendix A

**Proof of Proposition 1.** If $\frac{w}{\theta }>c_{n}$, $q_{n}=\frac{1-c_{n}}{2},q_{r}=0$; If $c_{n}\geq \frac{w}{\theta }\geq \frac{2c_{n}-1+\theta }{1+\theta }$, $q_{n}=\frac{1-c_{n}-\theta +w}{2\left(1-\theta \right)},q_{r}=\frac{c_{n}\theta -w}{2\theta -2\theta^{2}}$; If $\frac{2c_{n}-1+\theta }{1+\theta }>\frac{w}{\theta }$, $q_{n}=\frac{1-c_{n}+\theta -w}{2+6\theta },q_{r}=\frac{1-c_{n}+\theta -w}{2+6\theta }$; $\pi_{IR}^{R}\left(w\right)=\left(w-c_{r}\right)q_{r}=\{\begin{array}{c} 0\\ \frac{\left(w-\theta c_{n}\right)\left(w-c_{r}\right)}{2\left(-1+\theta \right)\theta }\\ \frac{\left(1-w+\theta -c_{n}\right)\left(w-c_{r}\right)}{2+6\theta } \end{array}$. Furthermore, we have If $c_{n}\leq \frac{c_{r}}{\theta }$, $w=\frac{c_{r}}{\theta }$, and $\pi_{IR}^{R}=0$. If $w=\frac{\theta c_{n}+c_{r}}{2}$, we have $\frac{2\left(1-\theta \right)\theta +\left(1+\theta \right)c_{r}}{\left(3-\theta \right)\theta }\geq c_{n}\geq \frac{c_{r}}{\theta }$ and $\pi_{IR}^{R}=\frac{{\left(\theta c_{n}-c_{r}\right)}^{2}}{8\left(1-\theta \right)\theta }$. If $w=\frac{1+\theta -c_{n}+c_{r}}{2}$, we have $c_{n}>\frac{1+\left(4-\theta \right)\theta +\left(1+\theta \right)c_{r}}{1+5\theta }$ and $\pi_{IR}^{R}=\frac{{\left(1+\theta -c_{n}-c_{r}\right)}^{2}}{8+24\theta }$. $\frac{{\left(\theta c_{n}-c_{r}\right)}^{2}}{8\left(1-\theta \right)\theta }<\frac{{\left(1+\theta -c_{n}-c_{r}\right)}^{2}}{8+24\theta }$, $c_{n}>{\hat{c}}_{n}=\frac{\left(1-\theta \right)\theta -\sqrt{\left(1-\theta \right)\theta \left(1+3\theta \right)}\left(\theta -c_{r}\right)-2\theta c_{r}}{\theta \left(1-3\theta \right)}$, and $c_{n}<\frac{\sqrt{\left(1-\theta \right)\theta }\left(1+\theta -c_{r}\right)+\sqrt{1+3\theta }c_{r}}{\theta \sqrt{1+3\theta }+\sqrt{\left(1-\theta \right)\theta }}=\frac{\left(1-\theta \right)\theta -\sqrt{\left(1-\theta \right)\theta \left(1+3\theta \right)}\left(\theta -c_{r}\right)-2\theta c_{r}}{\theta \left(1-3\theta \right)}$. Proposition 1 is proved. □

**Proof of Proposition 2.** First, we can obtain the best responses of the firm and the IR, which are given as follows: $q_{n}\left(q_{r}\right)=\frac{1-c_{n}-\left(1+\alpha \right)\theta q_{r}}{2}$ and $q_{r}\left(q_{n}\right)=\frac{\left(1-\alpha \right)\theta \left(1-q_{n}\right)-c_{r}}{2\left(1-\alpha \right)\theta }$. If $q_{r}\left(q_{n}\right)=\frac{\left(1-\alpha \right)\theta \left(1-q_{n}\right)-c_{r}}{2\left(1-\alpha \right)\theta }\leq 0$, $q_{r}=0$ and $q_{n}=\frac{1-c_{n}}{2}$; from $\frac{\left(1-\alpha \right)\theta \left(1-q_{n}\right)-c_{r}}{2\left(1-\alpha \right)\theta }\leq 0$, we have $c_{n}\leq \frac{2c_{r}-\left(1-\alpha \right)\theta }{\left(1-\alpha \right)\theta }$; If $0<q_{r}\left(q_{n}\right)=\frac{\left(1-\alpha \right)\theta \left(1-q_{n}\right)-c_{r}}{2\left(1-\alpha \right)\theta }<q_{n}$, $q_{r}=\frac{\left(1-\alpha \right)\theta \left(1+c_{n}\right)-2c_{r}}{\left(1-\alpha \right)\theta \left(4-\theta -\alpha \theta \right)}$ and $q_{n}=\frac{\left(1-\alpha \right)\left(2-\theta -\alpha \theta -2c_{n}\right)-\left(1+\alpha \right)c_{r}}{\left(1-\alpha \right)\theta \left(4-\theta -\alpha \theta \right)}$; If $q_{r}\left(q_{n}\right)=\frac{\left(1-\alpha \right)\theta \left(1-q_{n}\right)-c_{r}}{2\left(1-\alpha \right)\theta }\geq q_{n}$, we have $q_{r}=q_{n}=\frac{1-c_{n}}{2+\theta +\alpha \theta }$. From $\frac{\left(1-\alpha \right)\theta \left(1-q_{n}\right)-c_{r}}{2\left(1-\alpha \right)\theta }\geq q_{n}$, we have $c_{n}>\frac{\left(1-\alpha \right)\theta -\left(1-\alpha^{2}\right)\theta^{2}+\left(2+\theta +\alpha \theta \right)c_{r}}{3\left(1-\alpha \right)\theta }$. Proposition 2 is proved. □

**Proof of Proposition 3.** Based on Proposition 2, by differentiating $q_{r}^{M*}$ and $q_{n}^{M*}$ with respect to α, we have

(1) If $c_{n}\leq {\bar{c}}_{n}\left(\alpha \right)$*, then* $\frac{dq_{r}^{M*}}{d\alpha }=0$ *and* $\frac{dq_{n}^{M*}}{d\alpha }=0$*;*
(2) If ${\bar{c}}_{n}\left(\alpha \right)\leq c_{n}\leq {\tilde{c}}_{n}\left(\alpha \right)$*, then* $\frac{dq_{r}^{M*}}{d\alpha }=\frac{{\left(1-\alpha \right)}^{2}\theta^{2}\left(1+c_{n}\right)-4\left(2-\alpha \theta \right)c_{r}}{{\left(1-\alpha \right)}^{2}\theta {\left(4-\theta -\alpha \theta \right)}^{2}}>0$ $1-\frac{2c_{r}+2\sqrt{c_{r}\left(\left(2-\theta \right)\left(1+c_{n}\right)+c_{r}\right)}}{\theta \left(1+c_{n}\right)}>\alpha$ *and* $\frac{dq_{n}^{M*}}{d\alpha }=-\frac{2{\left(1-\alpha \right)}^{2}\theta \left(1+c_{n}\right)+\left(8-{\left(1+\alpha \right)}^{2}\theta \right)c_{r}}{{\left(1-\alpha \right)}^{2}\theta {\left(4-\theta -\alpha \theta \right)}^{2}}<0$*;*
(3) If $c_{n}>{\tilde{c}}_{n}\left(\alpha \right)$*, then* $\frac{dq_{r}^{M*}}{d\alpha }=\frac{dq_{n}^{M*}}{d\alpha }=-\frac{\theta \left(1-c_{n}\right)}{{\left(2+\theta +\alpha \theta \right)}^{2}}<0$.

Proposition 3 is proved. □

**Proof of Proposition 4.** According to Propositions 1 and 2, we have $\frac{c_{r}}{\theta }-\frac{2c_{r}}{\left(1-\alpha \right)\theta }+1=\frac{\left(1-\alpha \right)\theta -\left(1+\alpha \right)c_{r}}{\left(1-\alpha \right)\theta }>0\Leftrightarrow \alpha <\frac{\theta -c_{r}}{\theta +c_{r}}$ and ${\hat{c}}_{n}-\frac{\left(1-\alpha \right)\theta -\left(1-\alpha^{2}\right)\theta^{2}+\left(2+\theta +\alpha \theta \right)c_{r}}{3\left(1-\alpha \right)\theta }>0\Leftrightarrow 3\left(1-\alpha \right)\theta {\hat{c}}_{n}>\left(1-\alpha \right)\theta -\left(1-\alpha^{2}\right)\theta^{2}+\left(2+\theta +\alpha \theta \right)c_{r}$. Let $F\left(\alpha \right)=3\left(1-\alpha \right)\theta {\hat{c}}_{n}-\left(1-\alpha \right)\theta +\left(1-\alpha^{2}\right)\theta^{2}-\left(2+\theta +\alpha \theta \right)c_{r}$*,* then we have $F\left(0\right)=3\theta {\hat{c}}_{n}-\theta +\theta^{2}-\left(2+\theta \right)c_{r}>0$ and $F\left(1\right)=-2\left(1+\theta \right)c_{r}<0$*,* where ${\hat{c}}_{n}=\frac{\sqrt{\left(1-\theta \right)\theta }\left(1+\theta -c_{r}\right)+\sqrt{1+3\theta }c_{r}}{\theta \sqrt{1+3\theta }+\sqrt{\left(1-\theta \right)\theta }}$. Since F(α) is differentiable and continuous when 0<α<1. Therefore, there exists a threshold $\tilde{\alpha }$ so that F(α)<0 when $\alpha >\tilde{\alpha }$ and F(α)>0 when $\alpha <\tilde{\alpha }$. In summary, ${\bar{c}}_{n}\left(\alpha \right)<\frac{c_{r}}{\theta }$ and ${\tilde{c}}_{n}\left(\alpha \right)<{\hat{c}}_{n}$ when $\alpha <\min\left\{\frac{\theta -c_{r}}{\theta +c_{r}},\tilde{\alpha }\right\}$. Proposition 4 is proved. □

**Proof of Proposition 5.** According to Propositions 1, 2 and 4, when ${\bar{c}}_{n}\left(\alpha \right)<c_{n}<\frac{c_{r}}{\theta }$ and $\alpha <\frac{\theta -c_{r}}{\theta +c_{r}}$, we have $q_{r}^{R*}=0$ and $q_{r}^{M*}=\frac{\left(1-\alpha \right)\theta \left(1+c_{n}\right)-2c_{r}}{\left(1-\alpha \right)\theta \left(4-\theta -\alpha \theta \right)}$. Therefore, $q_{r}^{R*}<q_{r}^{M*}$. When $\max\left\{{\bar{c}}_{n}\left(\alpha \right),c_{r}/\theta \right\}<c_{n}<\min\left\{{\tilde{c}}_{n}\left(\alpha \right),{\hat{c}}_{n}\right\}$, we have $q_{r}^{R*}=\frac{\theta c_{n}-c_{r}}{4\theta \left(1-\theta \right)}$ and $q_{r}^{M*}=\frac{\left(1-\alpha \right)\theta \left(1+c_{n}\right)-2c_{r}}{\left(1-\alpha \right)\theta \left(4-\theta -\alpha \theta \right)}$, comparing $q_{r}^{R*}$ with $q_{r}^{M*}$, we get $q_{r}^{R*}-q_{r}^{M*}=-\frac{\left(1-\alpha \right)\theta \left(4-4\theta -\left(3-\alpha \right)\theta c_{n}\right)-\left(4\left(1-\alpha \right)-\left(7+\alpha^{2}\right)\theta \right)c_{r}}{4\left(1-\alpha \right)\left(1-\theta \right)\theta \left(4-\theta -\alpha \theta \right)}$. Thus, we have $q_{r}^{R*}<q_{r}^{M*}$ when $c_{n}<\frac{\left(1-\alpha \right)\theta \left(4-4\theta \right)-\left(4\left(1-\alpha \right)-\left(7+\alpha^{2}\right)\theta \right)c_{r}}{\left(3-\alpha \right)\left(1-\alpha \right)\theta^{2}}$. Furthermore, we have ${\tilde{c}}_{n}\left(\alpha \right)<c_{n}<\min\left\{{\hat{c}}_{n},\frac{4\left(1-\theta \right)\theta +\left(2+\theta +\alpha \theta \right)c_{r}}{\theta \left(6-\left(3-\alpha \right)\theta \right)c_{n}}\right\}$ and $\alpha <\tilde{\alpha }$, we have $q_{r}^{R*}=\frac{\theta c_{n}-c_{r}}{4\theta \left(1-\theta \right)}$ and $q_{r}^{M*}=\frac{1-c_{n}}{2+\theta +\alpha \theta }$. Similarly, we can obtain $q_{r}^{R*}-q_{r}^{M*}<0\Leftrightarrow c_{n}<\frac{4\left(1-\theta \right)\theta +\left(2+\theta +\alpha \theta \right)c_{r}}{\theta \left(6-\left(3-\alpha \right)\theta \right)c_{n}}$. Lastly, when $\max\left\{{\tilde{c}}_{n}\left(\alpha \right),{\hat{c}}_{n}\right\}<c_{n}$, we have $q_{r}^{R*}=\frac{1+\theta -c_{n}-c_{r}}{4\left(1+3\theta \right)}$ and $q_{r}^{M*}=\frac{1-c_{n}}{2+\theta +\alpha \theta }$. Moreover, we get $q_{r}^{R*}-q_{r}^{M*}>0\Leftrightarrow c_{n}>\frac{2+\theta \left(9-\alpha -\theta -\alpha \theta \right)+\left(2+\theta +\alpha \theta \right)c_{r}}{2+\left(11-\alpha \right)\theta }$. Proposition 5 is proved. □

**Proof of Proposition 6.** According to Propositions 1 and 2, we have $\pi_{IR}^{M}=\{\begin{array}{c} 0\\ \frac{{\left(\left(1-\alpha \right)\theta \left(1+c_{n}\right)-2c_{r}\right)}^{2}}{\left(1-\alpha \right)\theta {\left(4-\theta -\alpha \theta \right)}^{2}}\\ \frac{\left(1-c_{n}\right)\left(\left(1-\alpha \right)\theta \left(\theta +\alpha \theta +2c_{n}\right)-\left(2+\theta +\alpha \theta \right)c_{r}\right)}{{\left(2+\theta +\alpha \theta \right)}^{2}} \end{array}$, $\pi_{F}^{M}=\{\begin{array}{c} \frac{{\left(1-c_{n}\right)}^{2}}{4}\\ \begin{array}{c} \frac{\left(2-\theta -\left(2-\alpha \theta \right)c_{n}+c_{r}\right)\left(\left(1-\alpha \right)\left(2-\theta -\alpha \theta -2c_{n}\right)+\left(1+\alpha \right)c_{r}\right)}{\left(1-\alpha \right){\left(4-\theta -\alpha \theta \right)}^{2}}+\frac{\alpha \left(\left(1-\alpha \right)\theta \left(1+c_{n}\right)-2c_{r}\right)\left(\left(1-\alpha \right)\theta \left(1+c_{n}\right)+\left(2-\theta -\alpha \theta \right)c_{r}\right)}{{\left(1-\alpha \right)}^{2}\theta {\left(4-\theta -\alpha \theta \right)}^{2}} \end{array}\\ \frac{\left(1-c_{n}\right)\left(1+\alpha \theta \left(1+\theta +\alpha \theta \right)-\left(1-\alpha \theta \right)c_{n}\right)}{{\left(2+\theta +\alpha \theta \right)}^{2}} \end{array}$, $\pi_{IR}^{R}=\{\begin{array}{c} 0\\ \frac{{\left(\theta c_{n}-c_{r}\right)}^{2}}{8\left(1-\theta \right)\theta }\\ \frac{{\left(1+\theta -c_{n}-c_{r}\right)}^{2}}{8+24\theta } \end{array}$, and $\pi_{F}^{R}=\{\begin{array}{c} \frac{{\left(1-c_{n}\right)}^{2}}{4}\\ \frac{4\left(1-\theta \right)\theta +\theta \left(4-3\theta \right)c_{n}^{2}+c_{r}^{2}-2\theta c_{n}\left(4-4\theta +c_{r}\right)}{16\left(1-\theta \right)\theta }\\ \frac{{\left(1+\theta -c_{n}-c_{r}\right)}^{2}}{16+48\theta } \end{array}$. Thus, when α→0, then $\pi_{IR}^{M}\left(\alpha \to 0\right)=\{\begin{array}{c} 0\\ \frac{{\left(\theta \left(1+c_{n}\right)-2c_{r}\right)}^{2}}{\theta {\left(4-\theta \right)}^{2}}\\ \frac{\left(1-c_{n}\right)\left(\theta \left(\theta +2c_{n}\right)-\left(2+\theta \right)c_{r}\right)}{{\left(2+\theta \right)}^{2}} \end{array}$ and $\pi_{F}^{M}\left(\alpha \to 0\right)=\{\begin{array}{c} \frac{{\left(1-c_{n}\right)}^{2}}{4}\\ \frac{{\left(2-\theta -2c_{n}+c_{r}\right)}^{2}}{{\left(4-\theta \right)}^{2}}\\ \frac{{\left(1-c_{n}\right)}^{2}}{{\left(2+\theta \right)}^{2}} \end{array}$. Comparing $\pi_{IR}^{M}\left(\alpha \to 0\right)$ and $\pi_{F}^{M}\left(\alpha \to 0\right)$ with $\pi_{IR}^{R}$ and $\pi_{F}^{R}$, respectively, we have $\frac{{\left(1-c_{n}\right)}^{2}}{{\left(2+\theta \right)}^{2}}>\frac{{\left(1+\theta -c_{n}-c_{r}\right)}^{2}}{16+48\theta }\Leftrightarrow \frac{1-c_{n}}{2+\theta }-\frac{1+\theta -c_{n}-c_{r}}{4\sqrt{1+3\theta }}>0\Leftrightarrow c_{n}<\frac{2+\theta \left(3+\theta \right)-4\sqrt{1+3\theta }-\left(2+\theta \right)c_{r}}{2+\theta -4\sqrt{1+3\theta }}$ and $\frac{\left(1-c_{n}\right)\left(\theta \left(\theta +2c_{n}\right)-\left(2+\theta \right)c_{r}\right)}{{\left(2+\theta \right)}^{2}}>\frac{{\left(1+\theta -c_{n}-c_{r}\right)}^{2}}{8+24\theta }\Leftrightarrow c_{n}<\frac{4+\theta \left(16+\left(25-11\theta \right)\theta \right)+2\sqrt{6}\sqrt{\theta {\left(2+\theta \right)}^{2}\left(1+3\theta \right){\left(\theta -c_{r}\right)}^{2}}+\left(2+\theta \right)\left(2+11\theta \right)c_{r}}{4+\theta \left(20+49\theta \right)}$. We can prove that $\frac{2+\theta \left(3+\theta \right)-4\sqrt{1+3\theta }-\left(2+\theta \right)c_{r}}{2+\theta -4\sqrt{1+3\theta }}>\max\left\{{\tilde{c}}_{n}\left(0\right),{\hat{c}}_{n}\right\}$ for any θ and $c_{r}\leq \theta$. Therefore, there exists a threshold of the commission rate $\hat{\alpha }$ and a cost threshold ${\tilde{c}}_{n}\left(\hat{\alpha }\right)\;$ so that both the firm and the IR can obtain higher profit under the marketplace model when ${\tilde{c}}_{n}\left(\hat{\alpha }\right)>c_{n}>\max\left\{{\tilde{c}}_{n}\left(\alpha \right),{\hat{c}}_{n}\right\}$ and $\alpha <\hat{\alpha }$. Proposition 6 is proved. □

**Proof of Proposition 7.** Let $p_{r}=m+w$, and according to $p_{n}=1-q_{n}-\theta q_{r}$, and $p_{r}=\theta \left(1-q_{n}-q_{r}\right)$, we have $q_{r}=1-q_{n}-\frac{\left(w+m\right)}{\theta }$. Based on the first-order derivative condition, we have $w^{RS}=\frac{-m+\theta +c_{r}-\theta q_{n}}{2}$. Since $p_{r}=m+w=\theta \left(1-q_{n}-q_{r}\right)$, we have $m^{RS}=\theta \left(1-q_{n}-2q_{r}\right)-c_{r}$. Substituting $w^{RS}$ and $m^{RS}$ into Equation (7), we have $\pi_{F}^{RS}\left(q_{n},q_{r}\right)=q_{n}\left(1-c_{n}-q_{n}-\theta q_{r}\right)-q_{r}\left(c_{r}-\theta \left(1-q_{n}-2q_{r}\right)\right)$, $0\leq q_{r}\leq q_{n}$. Similar to the proof Proposition 1, we can obtain the result in Proposition 7. Proposition 7 is proved. □

**Proof of Proposition 8.** By using the backward induction approach, we first derive the IR’s optimal decision when given $q_{n}$. First, the IR’s best response function is $q_{r}\left(q_{n}\right)=\frac{\left(1-\alpha \right)\theta \left(1-q_{n}\right)-c_{r}}{2\left(1-\alpha \right)\theta }$. Therefore, if $q_{r}\left(q_{n}\right)=\frac{\left(1-\alpha \right)\theta \left(1-q_{n}\right)-c_{r}}{2\left(1-\alpha \right)\theta }\leq 0$, i.e., $1-\frac{c_{r}}{\left(1-\alpha \right)\theta }\leq q_{n}$, then $q_{r}^{*}\left(q_{n}\right)=0$. If $0<q_{r}\left(q_{n}\right)=\frac{\left(1-\alpha \right)\theta \left(1-q_{n}\right)-c_{r}}{2\left(1-\alpha \right)\theta }<q_{n}$, i.e., $\frac{1}{3}-\frac{c_{r}}{3\left(1-\alpha \right)\theta }<q_{n}<1-\frac{c_{r}}{\left(1-\alpha \right)\theta }$, then $q_{r}^{*}\left(q_{n}\right)=\frac{\left(1-\alpha \right)\theta \left(1-q_{n}\right)-c_{r}}{2\left(1-\alpha \right)\theta }$. If $q_{r}\left(q_{n}\right)=\frac{\left(1-\alpha \right)\theta \left(1-q_{n}\right)-c_{r}}{2\left(1-\alpha \right)\theta }\geq q_{n}$, i.e., $\frac{1}{3}-\frac{c_{r}}{3\left(1-\alpha \right)\theta }\geq q_{n}$, then $q_{r}^{*}\left(q_{n}\right)=q_{n}$. Let us turn to the firm’s objective function. By substituting $q_{r}^{*}\left(q_{n}\right)$ into Equation (9), we have the following sub-optimization problems:

(A1) $$\pi_{F}^{MS}\left(q_{n}\right)=\left(1-q_{n}-c_{n}\right))q_{n},\;s.t.\;1-\frac{c_{r}}{\left(1-\alpha \right)\theta }\leq q_{n}.$$

(A2) $$\pi_{F}^{MS}\left(q_{n}\right)=\frac{2\left(1-\alpha \right)\theta c_{r}q_{n}-\alpha c_{r}^{2}+{\left(1-\alpha \right)}^{2}\theta \left(\alpha \theta +q_{n}\left(4-2\left(1+\alpha \right)\theta -4c_{n}-\left(4-\left(2+\alpha \right)\theta \right)q_{n}\right)\right)}{4{\left(1-\alpha \right)}^{2}\theta },\;\text{s}.\text{t}.\;\frac{1}{3}-\frac{c_{r}}{3\left(1-\alpha \right)\theta }<q_{n}<1-\frac{c_{r}}{\left(1-\alpha \right)\theta }.$$

(A3) $$\pi_{F}^{MS}\left(q_{n}\right)=q_{n}\left(1+\alpha \theta -c_{n}-\left(1+\theta +2\alpha \theta \right)q_{n}\right),\;\;s.t.\;\frac{1}{3}-\frac{c_{r}}{3\left(1-\alpha \right)\theta }\geq q_{n}.$$

For the optimization problem (A1), we have $q_{n}^{*}=\frac{1-c_{n}}{2}$. From $1-\frac{c_{r}}{\left(1-\alpha \right)\theta }\leq q_{n}$, we have $c_{n}\leq \frac{2c_{r}}{\left(1-\alpha \right)\theta }-1$. $\pi_{F}^{MS}\left(q_{n}^{*}=\frac{1-c_{n}}{2}\right)=\frac{{\left(1-c_{n}\right)}^{2}}{4}$ and $\pi_{IR}^{MS}\left(q_{r}^{*}=0\right)=0$. For the optimization problem (A2), we have $q_{n}^{*}=\frac{\left(1-\alpha \right)\left(2-\theta -\alpha \theta -2c_{n}\right)+c_{r}}{\left(1-\alpha \right)\left(4-\left(2+\alpha \right)\theta \right)}$. From $\frac{1}{3}-\frac{c_{r}}{3\left(1-\alpha \right)\theta }<q_{n}<1-\frac{c_{r}}{\left(1-\alpha \right)\theta }$, we have $\frac{\left(4-\theta -\alpha \theta \right)c_{r}-\left(1-\alpha \right)\left(2-\theta \right)\theta }{2\left(1-\alpha \right)\theta }<c_{n}<\frac{\left(1-\alpha \right)\theta \left(2-\theta -2\alpha \theta \right)+\left(4+\left(1-\alpha \right)\theta \right)c_{r}}{6\left(1-\alpha \right)\theta }$. $\pi_{F}^{MS}\left(q_{n}^{*}=\frac{\left(1-\alpha \right)\left(2-\theta -\alpha \theta -2c_{n}\right)+c_{r}}{\left(1-\alpha \right)\left(4-\left(2+\alpha \right)\theta \right)}\right)=\frac{\left({\left(1-\alpha \right)}^{2}\theta \left({\left(2-\theta \right)}^{2}-4c_{n}\left(2-\theta -\alpha \theta -c_{n}\right)\right)+2\left(1-\alpha \right)\theta \left(2-\theta -\alpha \theta -2c_{n}\right)c_{r}-\left(4\alpha -{\left(1+\alpha \right)}^{2}\theta \right)c_{r}^{2}\right)}{4{\left(1-\alpha \right)}^{2}\theta \left(4-\left(2+\alpha \right)\theta \right)}$ and $\pi_{IR}^{MS}\left(q_{r}^{*}=\frac{\left(1-\alpha \right)\theta \left(2-\theta +2c_{n}\right)-\left(4-\theta -\alpha \theta \right)c_{r}}{2\left(1-\alpha \right)\theta \left(4-\left(2+\alpha \right)\theta \right)}\right)=\frac{{\left(\left(1-\alpha \right)\theta \left(2-\theta +2c_{n}\right)-\left(4-\theta -\alpha \theta \right)c_{r}\right)}^{2}}{4\left(1-\alpha \right)\theta {\left(4-\left(2+\alpha \right)\theta \right)}^{2}}$. For the optimization problem (A3), we have $q_{n}^{*}=\frac{1+\alpha \theta -c_{n}}{2+2\theta +4\alpha \theta }$. From $\frac{1}{3}-\frac{c_{r}}{3\left(1-\alpha \right)\theta }\geq q_{n}$, we have $c_{n}\geq \frac{\left(1-\alpha \right)\theta \left(1-\left(2+\alpha \right)\theta \right)+2\left(1+\theta +2\alpha \theta \right)c_{r}}{3\left(1-\alpha \right)\theta }$. $\pi_{F}^{MS}\left(q_{n}^{*}=\frac{1+\alpha \theta -c_{n}}{2+2\theta +4\alpha \theta }\right)=\frac{{\left(1+\alpha \theta -c_{n}\right)}^{2}}{4\left(1+\theta +2\alpha \theta \right)}$ and $\pi_{IR}^{MS}\left(q_{r}^{*}=\frac{1+\alpha \theta -c_{n}}{2+2\theta +4\alpha \theta }\right)=\frac{\left(1+\alpha \theta -c_{n}\right)\left(\left(1-\alpha \right)\theta \left(\theta +\alpha \theta +c_{n}\right)-\left(1+\theta +2\alpha \theta \right)c_{r}\right)}{2{\left(1+\theta +2\alpha \theta \right)}^{2}}$. Furthermore, we can obtain the following relationships: $\frac{2c_{r}}{\left(1-\alpha \right)\theta }-1-\frac{\left(4-\theta -\alpha \theta \right)c_{r}-\left(1-\alpha \right)\left(2-\theta \right)\theta }{2\left(1-\alpha \right)\theta }=-\frac{\theta \left(1-\alpha \right)-\left(1+\alpha \right)c_{r}}{2\left(1-\alpha \right)}$ and $\frac{\left(1-\alpha \right)\theta \left(1-\left(2+\alpha \right)\theta \right)+2\left(1+\theta +2\alpha \theta \right)c_{r}}{3\left(1-\alpha \right)\theta }-\frac{\left(1-\alpha \right)\theta \left(2-\theta -2\alpha \theta \right)+\left(4+\left(1-\alpha \right)\theta \right)c_{r}}{6\left(1-\alpha \right)\theta }=\frac{\left(1+3\alpha \right)c_{r}}{2\left(1-\alpha \right)}-\frac{\theta }{2}$. $-\frac{\theta \left(1-\alpha \right)-\left(1+\alpha \right)c_{r}}{2\left(1-\alpha \right)}>0\Leftrightarrow \alpha >\frac{\theta -c_{r}}{\theta +c_{r}}$ and $\frac{\left(1+3\alpha \right)c_{r}}{2\left(1-\alpha \right)}-\frac{\theta }{2}<0\Leftrightarrow \alpha <\frac{\theta -c_{r}}{\theta +3c_{r}}$. Thus, we can summarize the firm’s decisions as follows

(1) If $\alpha >\frac{\theta -c_{r}}{\theta +c_{r}}$, we have (i) If $c_{n}\leq {\overset{=}{c}}_{n}\left(\alpha \right)$, $q_{n}^{*}=\frac{1-c_{n}}{2}$; (ii) If ${\overset{=}{c}}_{n}\left(\alpha \right)<c_{n}<\frac{\left(1-\alpha \right)\theta \left(2-\theta -2\alpha \theta \right)+\left(4+\left(1-\alpha \right)\theta \right)c_{r}}{6\left(1-\alpha \right)\theta }$, $q_{n}^{*}=\frac{\left(1-\alpha \right)\left(2-\theta -\alpha \theta -2c_{n}\right)+c_{r}}{\left(1-\alpha \right)\left(4-\left(2+\alpha \right)\theta \right)}$; (iii) if
  , $q_{n}^{*}=\frac{1}{3}-\frac{c_{r}}{3\left(1-\alpha \right)\theta }$; (iv) If $c_{n}\geq \frac{\left(1-\alpha \right)\theta \left(1-\left(2+\alpha \right)\theta \right)+2\left(1+\theta +2\alpha \theta \right)c_{r}}{3\left(1-\alpha \right)\theta }$, $q_{n}^{*}=\frac{1+\alpha \theta -c_{n}}{2+2\theta +4\alpha \theta }$, where ${\overset{=}{c}}_{n}\left(\alpha \right)$ satisfies $\pi_{F}^{MS}\left(q_{n}^{*}=\frac{1-c_{n}}{2}\right)=\pi_{F}^{MS}\left(q_{n}^{*}=\frac{\left(1-\alpha \right)\left(2-\theta -\alpha \theta -2c_{n}\right)+c_{r}}{\left(1-\alpha \right)\left(4-\left(2+\alpha \right)\theta \right)}\right)$.
(2) If $\frac{\theta -c_{r}}{\theta +c_{r}}\geq \alpha \geq \frac{\theta -c_{r}}{\theta +3c_{r}}$, we have (i) If $c_{n}\leq \frac{2c_{r}}{\left(1-\alpha \right)\theta }-1$, $q_{n}^{*}=\frac{1-c_{n}}{2}$; (ii) If $\frac{2c_{r}}{\left(1-\alpha \right)\theta }-1<c_{n}<\frac{\left(4-\theta -\alpha \theta \right)c_{r}-\left(1-\alpha \right)\left(2-\theta \right)\theta }{2\left(1-\alpha \right)\theta }$, $q_{n}^{*}=1-\frac{c_{r}}{\left(1-\alpha \right)\theta }$; (iii) if $\frac{\left(4-\theta -\alpha \theta \right)c_{r}-\left(1-\alpha \right)\left(2-\theta \right)\theta }{2\left(1-\alpha \right)\theta }\leq c_{n}\leq \frac{\left(1-\alpha \right)\theta \left(2-\theta -2\alpha \theta \right)+\left(4+\left(1-\alpha \right)\theta \right)c_{r}}{6\left(1-\alpha \right)\theta }$, $q_{n}^{*}=\frac{\left(1-\alpha \right)\left(2-\theta -\alpha \theta -2c_{n}\right)+c_{r}}{\left(1-\alpha \right)\left(4-\left(2+\alpha \right)\theta \right)}$; (iv) If $\frac{\left(1-\alpha \right)\theta \left(2-\theta -2\alpha \theta \right)+\left(4+\left(1-\alpha \right)\theta \right)c_{r}}{6\left(1-\alpha \right)\theta }<c_{n}<\frac{\left(1-\alpha \right)\theta \left(1-\left(2+\alpha \right)\theta \right)+2\left(1+\theta +2\alpha \theta \right)c_{r}}{3\left(1-\alpha \right)\theta }$, $q_{n}^{*}=\frac{1}{3}-\frac{c_{r}}{3\left(1-\alpha \right)\theta }$; (v) If $c_{n}\geq \frac{\left(1-\alpha \right)\theta \left(1-\left(2+\alpha \right)\theta \right)+2\left(1+\theta +2\alpha \theta \right)c_{r}}{3\left(1-\alpha \right)\theta }$, $q_{n}^{*}=\frac{1+\alpha \theta -c_{n}}{2+2\theta +4\alpha \theta }$.
(3) If $\alpha <\frac{\theta -c_{r}}{\theta +3c_{r}}$, we have (i) If $c_{n}\leq \frac{2c_{r}}{\left(1-\alpha \right)\theta }-1$, $q_{n}^{*}=\frac{1-c_{n}}{2}$; (ii) If $\frac{2c_{r}}{\left(1-\alpha \right)\theta }-1<c_{n}<\frac{\left(4-\theta -\alpha \theta \right)c_{r}-\left(1-\alpha \right)\left(2-\theta \right)\theta }{2\left(1-\alpha \right)\theta }$, $q_{n}^{*}=1-\frac{c_{r}}{\left(1-\alpha \right)\theta }$; (iii) if $\frac{\left(4-\theta -\alpha \theta \right)c_{r}-\left(1-\alpha \right)\left(2-\theta \right)\theta }{2\left(1-\alpha \right)\theta }\leq c_{n}\leq {\tilde{\bar{c}}}_{n}\left(\alpha \right)$, $q_{n}^{*}=\frac{\left(1-\alpha \right)\left(2-\theta -\alpha \theta -2c_{n}\right)+c_{r}}{\left(1-\alpha \right)\left(4-\left(2+\alpha \right)\theta \right)}$; (iv) If ${\tilde{\bar{c}}}_{n}\left(\alpha \right)<c_{n}$, $q_{n}^{*}=\frac{1+\alpha \theta -c_{n}}{2+2\theta +4\alpha \theta }$, where ${\tilde{\bar{c}}}_{n}\left(\alpha \right)$ satisfies $\pi_{F}^{MS}\left(q_{n}^{*}=\frac{1+\alpha \theta -c_{n}}{2+2\theta +4\alpha \theta }\right)=\pi_{F}^{MS}\left(q_{n}^{*}=\frac{\left(1-\alpha \right)\left(2-\theta -\alpha \theta -2c_{n}\right)+c_{r}}{\left(1-\alpha \right)\left(4-\left(2+\alpha \right)\theta \right)}\right)$.

Proposition 8 is proved. □

**Proof of Proposition 9.** According to Propositions 7 and 8, when $\max\left\{{\tilde{\bar{c}}}_{n}\left(\alpha \right),\frac{\left(1-\alpha \right)\theta \left(1-\left(2+\alpha \right)\theta \right)+2\left(1+\theta +2\alpha \theta \right)c_{r}}{3\left(1-\alpha \right)\theta },\frac{\left(1+\theta \right)c_{r}+\left(2-\theta \right)\theta }{3\theta }\right\}<c_{n}$, we have $\pi_{F}^{MS}=\frac{{\left(1+\alpha \theta -c_{n}\right)}^{2}}{4\left(1+\theta +2\alpha \theta \right)}$, $\pi_{IR}^{MS}=\frac{\left(1+\alpha \theta -c_{n}\right)\left(\left(1-\alpha \right)\theta \left(\theta +\alpha \theta +c_{n}\right)-\left(1+\theta +2\alpha \theta \right)c_{r}\right)}{2{\left(1+\theta +2\alpha \theta \right)}^{2}}$, $\pi_{IR}^{R}=\frac{{\left(1+\theta -c_{n}-c_{r}\right)}^{2}}{8+24\theta }$, and $\pi_{F}^{R}=\frac{{\left(1+\theta -c_{n}-c_{r}\right)}^{2}}{16+48\theta }$. Thus, when α→0, then $\pi_{F}^{MS}\left(\alpha \to 0\right)=\frac{{\left(1-c_{n}\right)}^{2}}{4\left(1+\theta \right)}$ and $\pi_{IR}^{MS}\left(\alpha \to 0\right)=\frac{\left(1-c_{n}\right)\left(\theta \left(\theta +c_{n}\right)-\left(1+\theta \right)c_{r}\right)}{{\left(1+\theta \right)}^{2}}$. Comparing $\pi_{F}^{MS}\left(\alpha \to 0\right)$ and $\pi_{F}^{MS}\left(\alpha \to 0\right)$ with $\pi_{IR}^{R}$ and $\pi_{F}^{R}$, respectively, we have $\frac{{\left(1-c_{n}\right)}^{2}}{4\left(1+\theta \right)}>\frac{{\left(1+\theta -c_{n}-c_{r}\right)}^{2}}{4+16\theta }\Leftrightarrow \frac{3\theta -\left(1+\theta \right)\left(\theta -c_{r}\right)}{3\theta }>c_{n}$ and $\frac{\left(1-c_{n}\right)\left(\theta \left(\theta +c_{n}\right)-\left(1+\theta \right)c_{r}\right)}{{\left(1+\theta \right)}^{2}}>\frac{\theta {\left(1+\theta -c_{n}-c_{r}\right)}^{2}}{{\left(2+8\theta \right)}^{2}}\Leftrightarrow \frac{\theta \left(3+\theta \left(17+\left(19-31\theta \right)\theta \right)\right)-2\left(1+\theta \right)\left(1+4\theta \right)\sqrt{1+7\theta \left(1+2\theta \right)}\left(\theta -c_{r}\right)+\left(1+\theta \right)\left(2+\theta \left(15+31\theta \right)\right)c_{r}}{\theta \left(5+\theta \left(34+65\theta \right)\right)}>c_{n}$. Therefore, there exists a threshold of the commission rate $\hat{\bar{\alpha }}$ and a cost threshold $<{\tilde{\bar{c}}}_{n}\left(\hat{\bar{\alpha }}\right)$ so that both the firm and the IR can obtain higher profit under the marketplace model when $\max\left\{{\tilde{\bar{c}}}_{n}\left(\alpha \right),\frac{\left(1-\alpha \right)\theta \left(1-\left(2+\alpha \right)\theta \right)+2\left(1+\theta +2\alpha \theta \right)c_{r}}{3\left(1-\alpha \right)\theta },\frac{\left(1+\theta \right)c_{r}+\left(2-\theta \right)\theta }{3\theta }\right\}<c_{n}<{\tilde{\bar{c}}}_{n}\left(\hat{\bar{\alpha }}\right)$ and $\alpha <\hat{\bar{\alpha }}$. Proposition 9 is proved. □
